# Respiratory Picornaviruses and Respiratory Syncytial Virus as Causative Agents of Acute Expiratory Wheezing in Children

**DOI:** 10.3201/eid1006.030629

**Published:** 2004-06

**Authors:** Tuomas Jartti, Pasi Lehtinen, Tytti Vuorinen, Riikka Österback, Bernadette van den Hoogen, Albert D.M.E. Osterhaus, Olli Ruuskanen

**Affiliations:** * Turku University Hospital, Turku, Finland;; † Turku University, Turku, Finland;; ‡ Erasmus Medical Center, Rotterdam, the Netherlands

**Keywords:** wheezing, bronchiolitis, asthma, rhinovirus, enterovirus, respiratory syncytial virus

## Abstract

We studied the viral etiology of acute expiratory wheezing (bronchiolitis, acute asthma) in 293 hospitalized children in a 2-year prospective study in Finland. A potential causative viral agent was detected in 88% of the cases. Eleven different viruses were represented. Respiratory syncytial virus (RSV) (27%), enteroviruses (25%), rhinovirus (24%), and nontypable rhino/enterovirus (16%) were found most frequently. In infants, RSV was found in 54% and respiratory picornaviruses (rhinovirus and enteroviruses) in 42% of the cases. In older children, respiratory picornaviruses dominated (65% of children ages 1-2 years and 82% of children ages >3 years). Human metapneumovirus was detected in 4% of all children and in 11% of infants. To prevent and treat acute expiratory wheezing illnesses in children, efforts should be focused on RSV, enterovirus, and rhinovirus infections.

Acute expiratory wheezing illnesses (bronchiolitis, acute asthma) are the primary causes of hospitalization in children. An estimated 3% of children without other medical conditions are hospitalized for bronchiolitis (*1*). The annual hospitalization rate for exacerbation of asthma is 0.15% in children (*2*). In the United States alone, ≈200,000 children are hospitalized for bronchiolitis and acute asthma each year, which causes a substantial impact on families and the community.

Respiratory viruses are the most important precipitants of acute expiratory wheezing in children (*3**,**4*). Bronchiolitis is reportedly induced in infants mainly by respiratory syncytial virus (RSV), and asthma in older children is induced mainly by rhinovirus. The role of rhinovirus in infants is not clear. Furthermore, the roles of other respiratory viruses, e.g., enteroviruses, and the recently discovered human metapneumovirus (HMPV) in the etiology of acute wheezing are not well established (*5**,**6*). Investigating the viral origin of acute expiratory wheezing is useful because some antiviral treatments and vaccination are available, and the efficacy of anti-inflammatory treatments may be related to viral origin.

The purpose of the study was to investigate the role of 11 respiratory viruses in children hospitalized for acute expiratory wheezing. The viral etiology was studied for 2 years prospectively to cover outbreaks of all major respiratory viruses. Virus culture, virus antigen detection, polymerase chain reaction (PCR) techniques, and serologic testing were used to optimize the diagnosis of viral infection.

## Methods

### Study Participants and Definitions

As part of a randomized clinical trial evaluating the efficacy of systemic corticosteroid in the treatment of acute expiratory wheezing in children, we investigated the viral etiology of the infections. From September 1, 2000, through May 31, 2002, a total of 293 children participated in the study in the Department of Pediatrics, Turku University Hospital. Study breaks occurred from June to July 2001 and during Christmas week 2001. Inclusion criteria were the following: age from 3 months to 16 years, hospitalization for acute expiratory wheezing, and written informed consent from the parents. Exclusion criteria were the following: chronic diseases other than asthma or allergy, systemic glucocorticoid treatment within 4 weeks before the study, severe wheezing necessitating intensive-care unit treatment, and previous participation in this study. The study protocol was approved by the Ethics Committee of the Turku University Hospital.

Acute expiratory wheezing was called bronchiolitis when it occurred in children <3 years of age. When it recurred >2 times in persons of any age or occurred in persons >3 years of age, the diagnosis of asthma was used (*7*). To some extent, bronchiolitis and asthma are expressions of the same pathologic process, and no rigid criteria separate these illnessess. All patients were examined by one of the two study physicians (T.J. and P.L.).

### Sample Collection

On patient's admission, a nasopharyngeal aspirate sample was taken through a nostril by inserting a disposable catheter connected to a mucus extractor to a depth of 5 to 7 cm and retracting it slowly while applying gentle suction with an electric suction device. All specimens were obtained without inserting any solution into the nostrils. Disposable plastic gloves were used, and all surfaces were wiped with disinfectant to prevent contamination. Immediately after the secretion was suctioned, two sterile cotton swabs were dipped in the aspirate. The swabs were then placed in vials containing 2 mL of viral transport medium (5% tryptose phosphate broth, 0.5% bovine serum albumin, and antimicrobial agents in phosphate-buffered saline) for virus culture and PCR assays. The rest of the mucus was used for virus antigen detection. The specimens were transported to the laboratory on the same day at room temperature. The tubes for RSV and HMPV PCR assays were frozen at –70°C before processing. Blood samples were collected on patient's admission and 2–3 weeks after discharge from the hospital.

### Virologic Methods

Viral antigens for adenovirus; influenza A and B viruses; parainfluenza virus types 1, 2, and 3; and RSV were detected by time-resolved fluoroimmunoassay (*8*). Immunoglobulin (Ig) G antibodies to the same viruses were measured from paired serum samples by enzyme immunoassays as described earlier (*9**–**11*). Purified heat-treated coxsackievirus A9, coxsackievirus B3, echovirus 11, and poliovirus 1 were used as an antigen mixture in enterovirus IgG assays and purified heat-treated coxsackievirus A16, coxsackievirus B3, and echovirus 11 in IgM assays (*12*). Virus culture was performed according to routine protocols in A549, HeLa, and LLC-Mk2 cell lines and human foreskin fibroblasts (*13*). The supernatants of cell cultures exhibiting a cytopathogenic effect were further studied by antigen detection for adenovirus; influenza A and B viruses; parainfluenza virus types 1, 2, and 3; and RSV or by reverse transcription (RT)-PCR for enteroviruses and rhinovirus. Nucleic acids for RT-PCR were isolated from the nasopharyngeal samples with a commercial kit (High Pure Viral Nucleic Acid Kit, Roche Diagnostics, Mannheim, Germany) according to the manufacturer's instructions. RT-PCR was used to detect enteroviruses and rhinovirus, coronavirus, RSV, and HMPV, as described previously (*6**,**14**,**15*). A case was defined as virus positive if at least one of the tests used was positive for virus. The rates of HMPV, respiratory picornaviruses, and RSV detected during the first study season 2000–2001 have been published (*16*).

### Statistical Methods

The chi-square test was used for intergroup comparisons of differrent age groups in specific virus groups. The results were analyzed by using SAS software (version 8.2, SAS Institute, Cary, NC).

## Results

### Patient Characteristics

**Figure 1:**
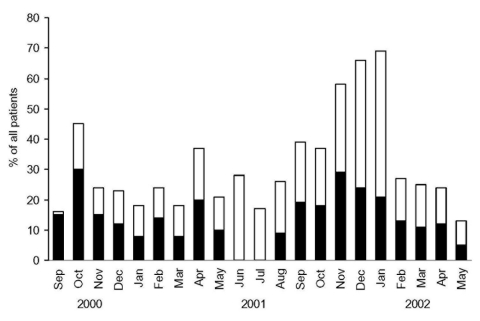
Hospitalized children with expiratory wheezing during the study period. Black indicates included patients.

From September 2000 through May 2002, a total of 661 children were hospitalized for acute expiratory wheezing (Figure 1). Of the 661 patients, 341 did not meet the study criteria: 87 had already participated in the study, 79 were <3 months of age, 55 were not enrolled during study breaks, 48 had had systemic glucocorticoid treatment within 4 weeks, 24 did not need hospitalization, 17 had a chronic disease, 12 had guardians with language difficulties, 11 needed treatment in our intensive care unit, 3 had guardians who were not present, 2 were exposed to varicella, 2 patients' cases were not reported to the study physician, and 1 child was not eligible because of social reasons. The remaining 320 were eligible, but the parents of 27 (8%) children did not give their consent for participation in the study. Eventually, 293 children participated in the study.

The median age of the 293 study patients was 1.6 years (range 3 months–15.2 years). Seventy-six (26%) children were <12 months of age, 152 (52%) children were 12–35 months old, and 65 (22%) children were >3 years. In 179 children, the clinical diagnosis was bronchiolitis and in 114, acute asthma. Of the children with asthma, 49 were <3 years of age, 53% were boys, 38% experienced atopy, and 41% had parents who smoked.

### Virus Infections

A potential causative viral agent was detected in 88% of the cases (Table 1). RSV (27%), enteroviruses (25%), and rhinovirus (24%) were the most common causative agents, resulting in 31%, 28%, and 28% of 258 virus-positive cases, respectively. The viruses in samples identified by the primary picornavirus PCR test but not identifiable in the liquid-hybridization assay were named rhino/enteroviruses (16%). According to our sequence data, these amplicons have shown >90% homology to human rhinoviruses. The remaining eight viruses studied accounted for 18% of the cases, and none of these viruses was detected in >5% of all cases.

**Table 1:** Positive viral findings in 293 children hospitalized for acute expiratory wheezing^a^

Virus	Virus antigen test; n = 293	Virus culture; n = 292	Virus PCR;^b^ n = 291	Virus serology; n = 266	Total; n = 293
Respiratory syncytial virus (RSV)	62 (21)	58 (20)	50 (18)	56 (21)	80 (27)
Enterovirus		14 (5)	59 (20)	27 (10)	72 (25)
Rhinovirus		25 (9)	65 (22)		71 (24)
Rhino/enterovirus		1 (0.3)	46 (16)		46 (16)
Parainfluenza virus type 1	8 (3)	0			8 (3)
Parainfluenza virus type 2	0	0		0	0
Parainfluenza virus type 3	4 (1)	1 (0.3)			5 (2)
Parainfluenza virus type 1 or 3				8 (3)	4 (1)
Adenovirus	0	9 (3)		6 (2)	15 (5)
Human metapneumovirus			12 (4)		12 (4)
Influenza A virus	1 (0.3)	1 (0.3)		2 (0.8)	3 (1)
Influenza B virus	4 (1)	2 (0.7)		2 (0.8)	4 (1)
Coronavirus		0	4 (1)		4 (1)
Mixed viral infection					57 (19)
Total	79 (27)	111 (38)	236 (81)	101 (38)	258 (88)

^a^Data are presented as number of samples positive (% of evaluated samples). ^b^For RSV polymerase chain reaction (PCR), n = 279.

Mixed viral infections were found in 57 (19%) cases and were usually associated with respiratory picornaviruses. Coinfection with enteroviruses and RSV was the most common mixed infection (19%), followed by rhinovirus and RSV (14%), rhino/enterovirus and RSV (11%), and enteroviruses and rhinovirus (9%). Of 12 HMPV infections, 5 were associated with other respiratory viruses.

Most of the viruses (84%), respiratory picornaviruses especially, were detected by using PCR (Table 1). Rhinovirus was cultivated in 25 (38%) of 65 specimens with PCR-positive results, enteroviruses in 14 (24%) of 59 specimens with PCR-positive results, and rhino/enterovirus in 1 (2%) of 46 specimens with PCR-positive results. To compare different methods of detecting RSV infection, we selected the patients whose samples were studied with four methods (n = 257). The recovery rate of RSV by IgG serologic testing was 22%; by virus antigen detection, 21%; by virus culture, 20%; and by PCR, 18%.

**Figure 2:**
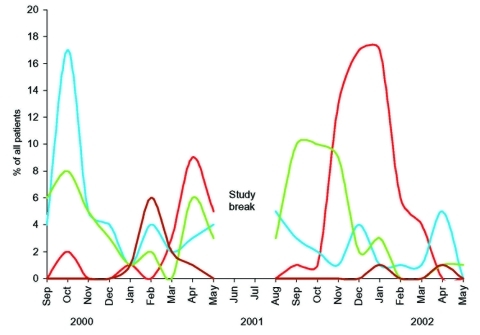
The epidemics of respiratory syncytial virus (red), rhinovirus (blue), enterovirus (green), and human metapneumovirus (brown) during the study period.

Typical of the situation in Finland, a minor RSV epidemic occurred during the spring of 2000, followed by a major epidemic during the winter of 2001 to 2002 (Figure 2). Enterovirus outbreaks were seen during the fall in both 2000 and 2001. Rhinovirus outbreaks occurred during fall and spring of both years. An HMPV epidemic was seen during the winter of 2001. HMPV was detected in 30% of the study children during the 3-month epidemic. During the peak 3 epidemic months of respiratory picornaviruses, from September to November 2000, they accounted for 82% of all cases, and only 5% had other viral causes. During the peak 3 epidemic months of RSV, from November 2001 through January 2002, RSV accounted for 65% of all cases, and other viruses were found in 20%. Influenza A virus epidemics occured in the community from the beginning of October 2000 to the end of March 2001 and from October 2001 to May 2002 (data not shown), but influenza A virus caused only three cases of acute expiratory wheezing for which the patient had to be hospitalized.

**Figure 3:**
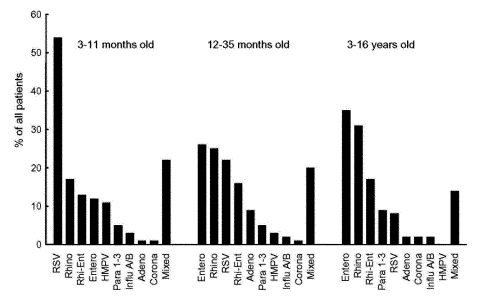
The prevalence of respiratory viruses in hospitalized, wheezing children in different age groups. RSV, respiratory syncytial virus; RHINO, rhinovirus; RHI-ENT, rhino/enterovirus; ENTERO, enteroviruses; HMPV, human metapneumovirus; PARA 1–3, parainfluenza virus types 1–3; INFLU A/B, influenza A and B viruses; ADENO, adenovirus; CORONA, coronavirus; MIXED, mixed viral infection. p values are for intergroup comparisons: RSV p < 0.001, HMPV p = 0.003, enteroviruses p = 0.0018, and adenovirus p = 0.022.

RSV (54%), respiratory picornaviruses (42%), and HMPV (11%) were the most common viruses in infants (Figure 3). Respiratory picornaviruses were detected in 65% and RSV in 22% of the cases in children ages 12–35 months. In children aged >3 years, respiratory picornaviruses (82%) were found most frequently.

Comparisons between age groups showed that RSV (p < 0.001) and HMPV (p = 0.0030) infected infants significantly more often than children in other age groups, adenovirus infected children ages 1–2 years significantly more often (p = 0.022), and enteroviruses infected children ages >3 years more often (p = 0.0018; Figure 3). No other significant differences were found.

##  Discussion

Our prospective study produced four notable findings. First, respiratory virus infection was detected in up to 90% of hospitalized children with acute expiratory wheezing. Second, respiratory picornaviruses were commonly associated with wheezing in infants. Third, one third of the wheezing children ages >3 years were infected with enteroviruses. Fourth, HMPV infections occurred in infants, but mainly during the first study year, and they were associated with only 4% of all cases with expiratory wheezing.

All major studies of the viral origins of expiratory wheezing are presented in Table 2. In studies from the 1960s to the 1990s, viral diagnosis was based on conventional virus culture, antigen detection, and serologic testing, and a viral agent could be established in 20% to 50% of children with expiratory wheezing. Bronchiolitis was mainly considered an RSV infection, with recovery in up to 73% of patients with confirmed cases (*30*). Lower RSV recovery rates were seen in older children (*17**,**18**,**21**,**22**,**24**,**27*). In the 1990s, viral detection rates increased to 75% to 85%, mainly as result of the increased detection of rhinoviruses by PCR (*3**,**5*). Our data confirm that RSV plays a key role in the etiology of bronchiolitis during RSV epidemics. Our findings regarding bronchiolitis give a prominent role also to rhinovirus, which has earlier been considered a common causative agent of wheezing in older children only (*3**,**4**,**28**,**31*). We found no differences in the distribution of rhinovirus infections by patient age.

**Table 2:** Viral etiology of acute expiratory wheezing in children^a,b^

Y of study	Wheezing episodes	Age (y)	Methods for virus detection				Viral identification rates (%)								
			Culture	Antigen	Serology	PCR	RSV	Rhino	Entero	PIV types 1–3	Influ A/B	Adeno	Corona	HMPV	Total pos.
1965 (17)	225	0–16	+				8	3	5	4	0	4			27
1971 (18)	855	0–14	+				9	2	1	8	1	2			25
1975 (19)	1,515	0–12	+				3	12	3	3	2	1			23
1976 (20)	267	1–12	+				4	6	1	1	1	3			14
1979 (21)	1,851	0–15	+				7	1		6		2			21
1979 (22)	554	0–12	+				2	13	4	4	2	1			26
1979 (23)	72	5–15	+				1	28		3	10	1			49
1984 (24)	256	2–15	+	+			5			4	2	3	2		29
1987 (25)	204	0–12	+	+			6	1	5	0	3	2			19
1993 (26)	99	0.2–16	+	+			14	19	3	0	2	0			36
1996 (27)	181	0.3–2	+	+			12	6	1	7		2			26
1999 (3)	70	0.2–16	+	+			26	61	1		1		4		83
1999 (28)	132	0.3–14		+			21	47	10	4	5	5	5		82
2000 (29)	84	0.7		+			54	10	8	0	0	13	0		74
2002 (30)	118	0–1.5					53	21		3	3	8	3		74
2003 (4)	179	0.1–17	+	+			7	79		1	2			2	88

^a^Sampling period of at least 12 mo. ^b^RSV, respiratory syncytial virus; PCR, polymerase chain reaction; rhino, rhinovirus; entero, enterovirus; PIV, parainfluenza virus; influ, influenza virus; adeno, adenovirus; corona, coronavirus; HMPV, human metapnemovirus; pos., positive.

This is the first long-term study to report a high association of enterovirus infections with acute expiratory wheezing in children. Enteroviruses, which replicate most prolifically in the gastrointestinal tract, have recently been shown to be associated with upper respiratory infections in 25% to 35% of the cases (*32**,**33*). Our findings are in agreement with those of Rawlinson et al. (*4*), who found enteroviruses by PCR in 29% of the young children with well-documented asthma during the summer. We found enteroviruses mostly in older children.

HMPV was detected in 4% of our patients. A recent study of children hospitalized for acute respiratory tract disease found HMPV in 6% of the cases (*34*). Bronchiolitis and pneumonitis were the main diagnoses. HMPV predominantly infected infants as seen in our study and in previous studies (*4**,**6*). HMPV outbreaks have been reported mainly in mid-winter, which was supported by our study. Notably, the HMPV outbreak with 10 cases was seen during the first study year, and two cases were found during the second year, which suggests that epidemics do not occur every year.

The use of PCR has markedly increased the recovery rates of viruses in acute respiratory infections (*3**,**5*). The clinical value of positive respiratory picornavirus PCR tests is, however, questionable as picornavirus RNA has also been detected in 5% to 30% of asymptomatic children (*3**,**35*). We recently found that the number of positive PCR results for picornavirus markedly decreased over 2 to 3 weeks and disappeared over 5 to 6 weeks after an acute respiratory infection, which suggests that a positive PCR result for picornaviruses is related to acute infection (*36*). None of the 79 healthy controls were infected with enteroviruses, but 16% were positive for rhinovirus or nontypeable rhino/enterovirus (*36*). In detecting RSV infections, PCR was no more sensitive than virus culture, antigen detection, or serologic testing. This finding is in contrast to the results of previous studies, especially in adults (*37*). In children too, PCR has been almost 1.5 times more sensitive than culture and antigen detection (*38*). These differences may be explained by the greater sensitivity of the nested RT-PCR used in those studies. Compared to children, adults may also have lower titers of viruses in the nasopharynx, which favors PCR diagnosis over virus culture or antigen detection. We likely did not miss many cases of adenovirus, parainfluenza virus, or influenza virus infections because PCR has only modestly increased sensitivity to those viruses compared to virus culture and antigen detection (*39**,**40*).

Our study has some limitations. First, and most important, we studied fewer than half of the children admitted to our hospital for acute expiratory wheezing. However, throughout the study, we enrolled approximately half of the patients hospitalized for expiratory wheezing each month. Since the respiratory virus season is the main factor determining the viral cause of acute illness, any seasonality bias is largely excluded. Several infants with RSV infection were missed, because infants <3 months of age were not included. However, during the summer study break, when rhinovirus and enteroviruses are normally circulating in the community, children were not enrolled. This balances the ratio of missed RSV cases to picornavirus cases. We therefore believe that our sample reliably represents the whole patient population hospitalized during the study years. Furthermore, we only analyzed viral infections. *Chlamydia pneumoniae* and *Mycoplasma pneumoniae* have been detected in 5% to 25% of children with acute wheezing, but the clinical importance of these findings remains to be determined (*7**,**28*).

In conclusion, this study showed that acute expiratory wheezing necessitating hospitalization was most often associated with RSV, enterovirus, and rhinovirus infections. Acute expiratory wheezing in infants may be a risk factor for childhood asthma (*31*). Therefore, efforts should focus on developing antiviral agents and vaccines against RSV and respiratory picornaviruses.
